# Supplementary material for: Urban development pattern’s influence on extreme rainfall occurrences
Source: Nat Commun. 2024 May 11;15:3997. doi: 10.1038/s41467-024-48533-5 (PMC11088619; doi:10.1038/s41467-024-48533-5)
Supplement: Supplementary file 1 — Supplementary Information [file 41467_2024_48533_MOESM1_ESM.pdf]

Supplementary information for

Urban development pattern’s influence on extreme rainfall occurrences

Content of SI

Supplementary Figures 1-17

Supplementary Tables 1-3

Supplementary Figures

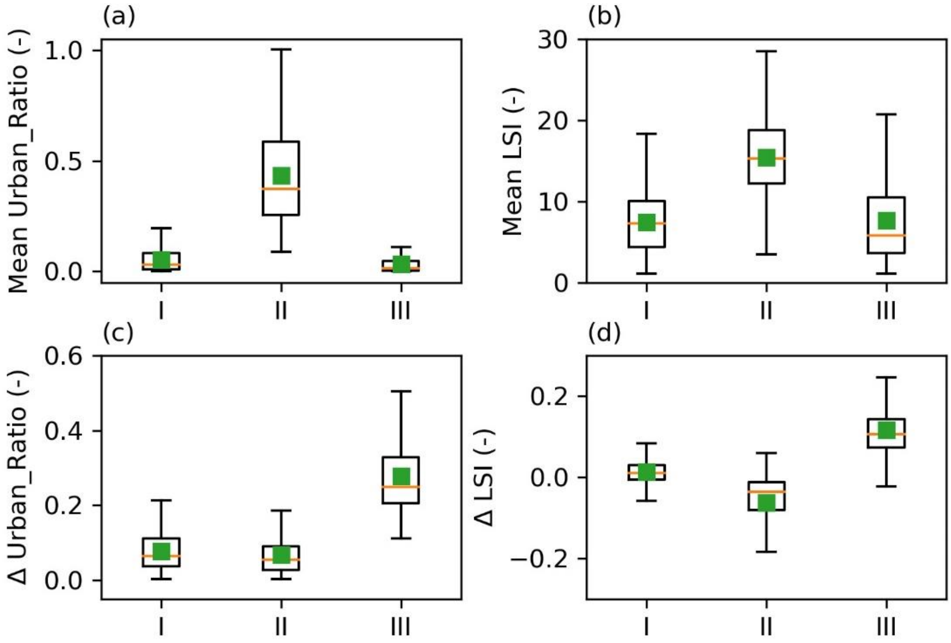

Supplementary Fig. 1 Comparisons across three city groups. Boxplots of (a) mean urban ratios, i.e., (TA_{2003} + TA_{2018})/2; (b) mean landscape shape index, i.e., (LSI_{2003} + LSI_{2018})/2; (c) changes in urban ratio, i.e., (TA_{2018} - TA_{2018})/ (TA_{2018} + TA_{2018}); (d) changes in landscape shape index, i.e., (LSI_{2018} - LSI_{2018})/ (LSI_{2018} + LSI_{2018}) for the cities of three different groups with contrast urban development patterns. The box spans the 25th and 75th percentiles, and the whiskers represents 5th and 95th percentiles. The red line represents the median values, while green squares show the mean values.

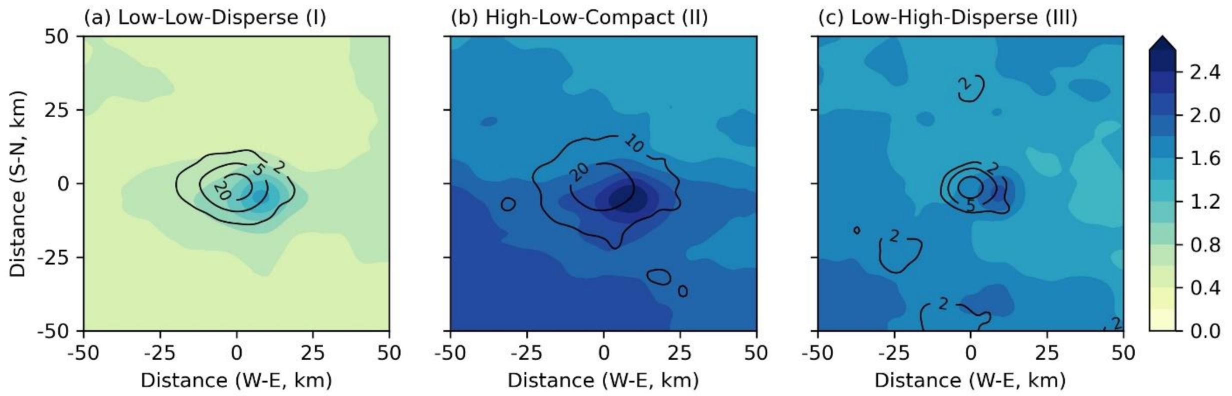

Supplementary Fig. 2 Contrasting rainfall patterns across three city groups. Composite mean change ratios in extreme rainfall (i.e., exceeding the 90th percentile daily rainfall of rainy days) frequencies for different city groups with diverse development patterns between the period 2000-2005 and 2016-2020. (a) Low-Low-Disperse city group, (b) High-Low-Compact city group, and (c) Low-High-Disperse city group. The contour shows composite mean number of urban pixels in the domain, highlighting the urban boundary.

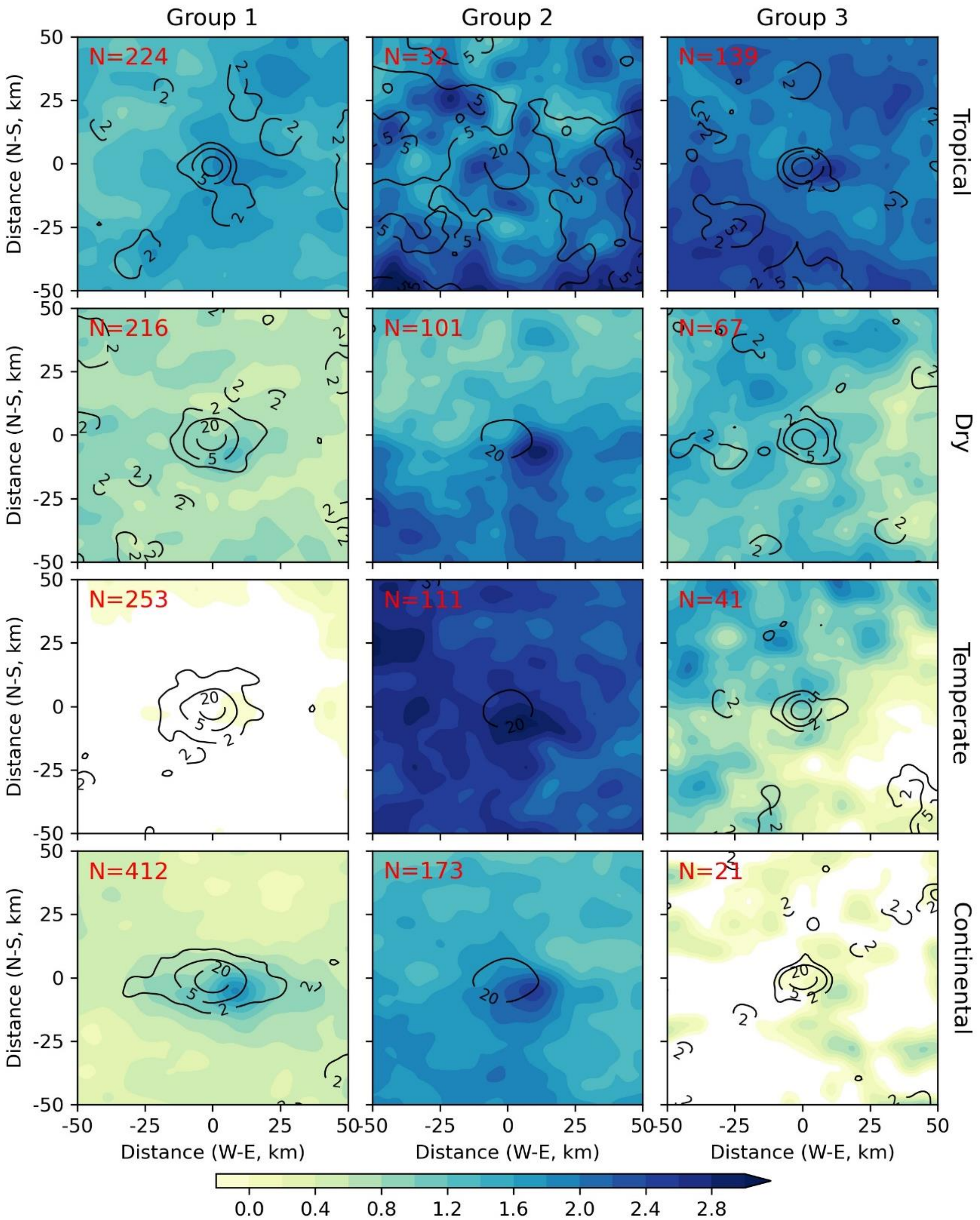

Supplementary Fig. 3 Contrasting rainfall patterns across different city groups and background climates (i.e., tropical, dry, temperate, and continental). The number shown in each subplot indicate number of cities belonging to each category. The contour shows composite mean number of urban pixels in the domain, highlighting the urban boundary. Note that the small number of cities may lead to large uncertainty in interpretating rainfall patterns, for instance, Group 2 in tropical climate and Group 3 in continental climate.

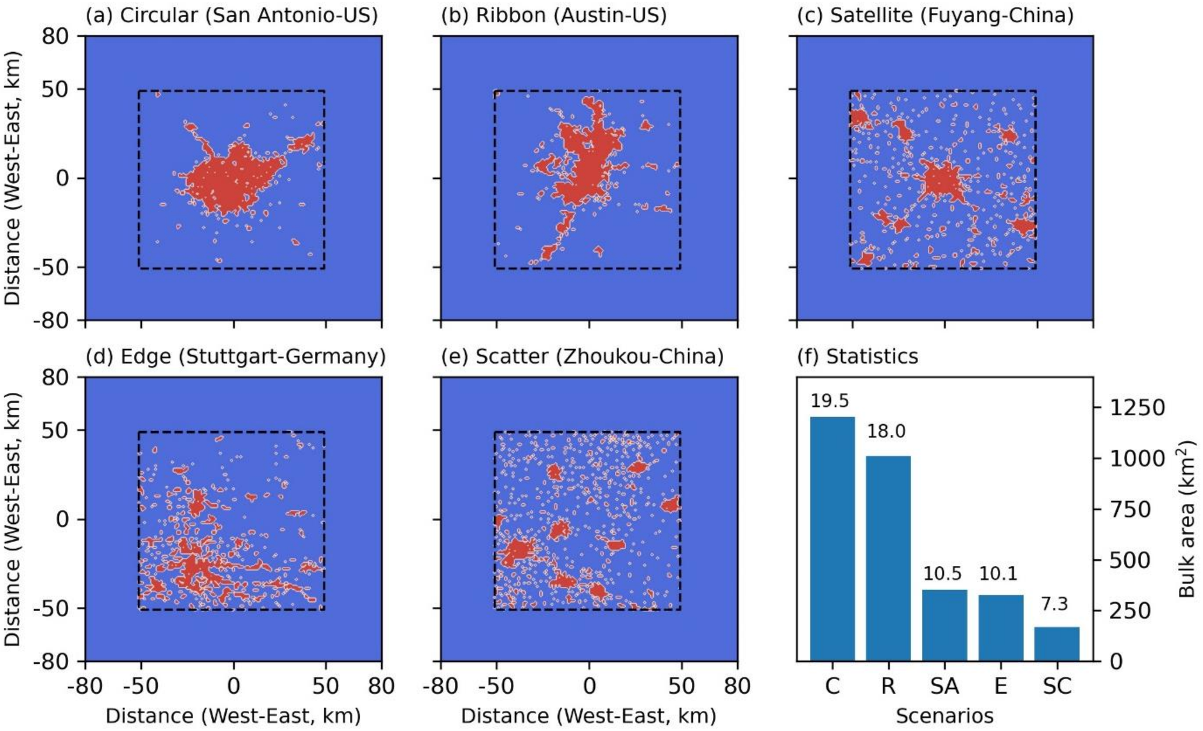

Supplementary Fig. 4 Layouts of five urban scenarios. (a-e) Spatial patterns of urban footprints for the five urban scenarios. The dashed box represents the 1 °×1 ° domain. The outer boundary of each plot shows the spatial extent of the innermost domain for the RAIL simulations; (f) Areas of the largest urban patch for each scenario. A patch is defined as a cluster of contiguous urban grids. The number on each bar is the equivalent radius (i.e., the square root of the patch area divided by 3.14) of the largest urban patch, as an index for city size.

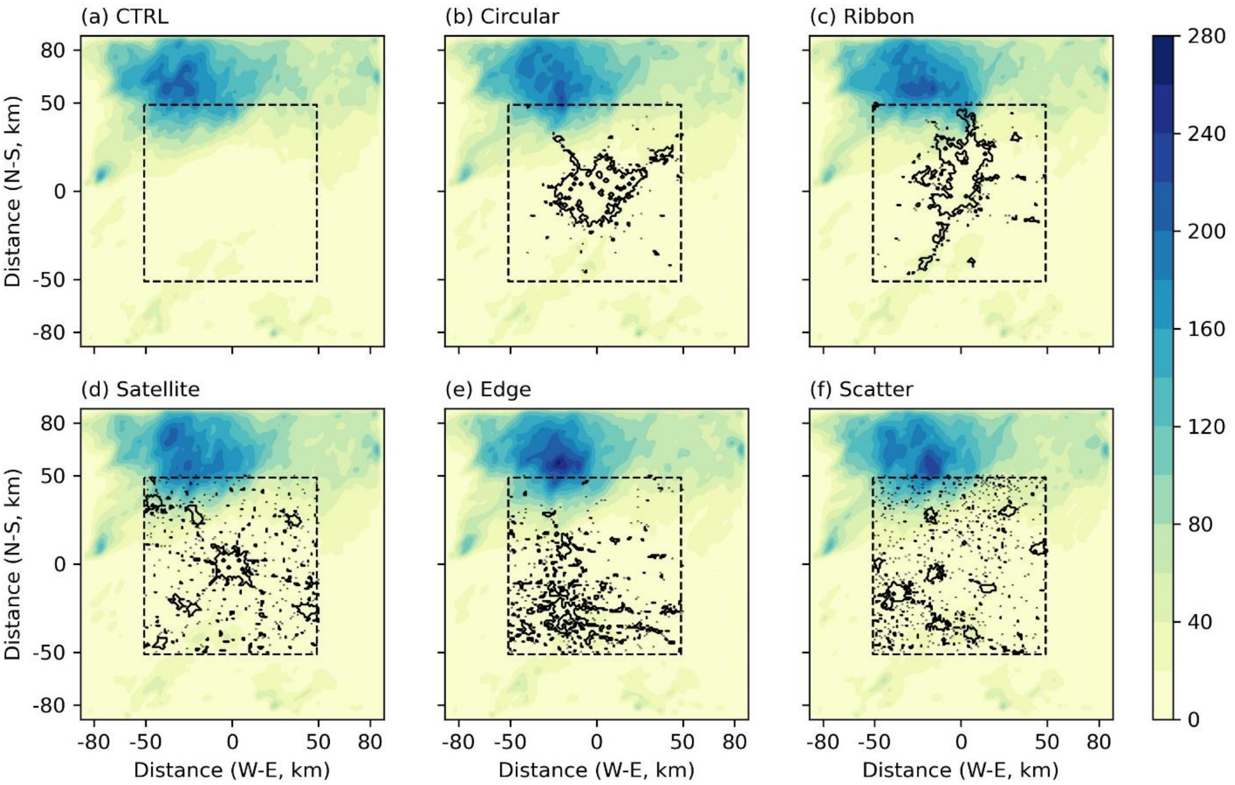

Supplementary Fig. 5 Spatial distribution of total rainfall (in mm) for the "no-city" scenario (CTRL) and five urban scenarios. The results represent ensemble mean of six-member simulations for each scenario. (a) CTRL (i.e., "no-city" scenario), (b) Circular, (c) Ribbon, (d) Satellite, (e) Edge, and (f) Scatter. The dashed box represents the 1 °×1 ° domain, while the contour highlights urban footprints.

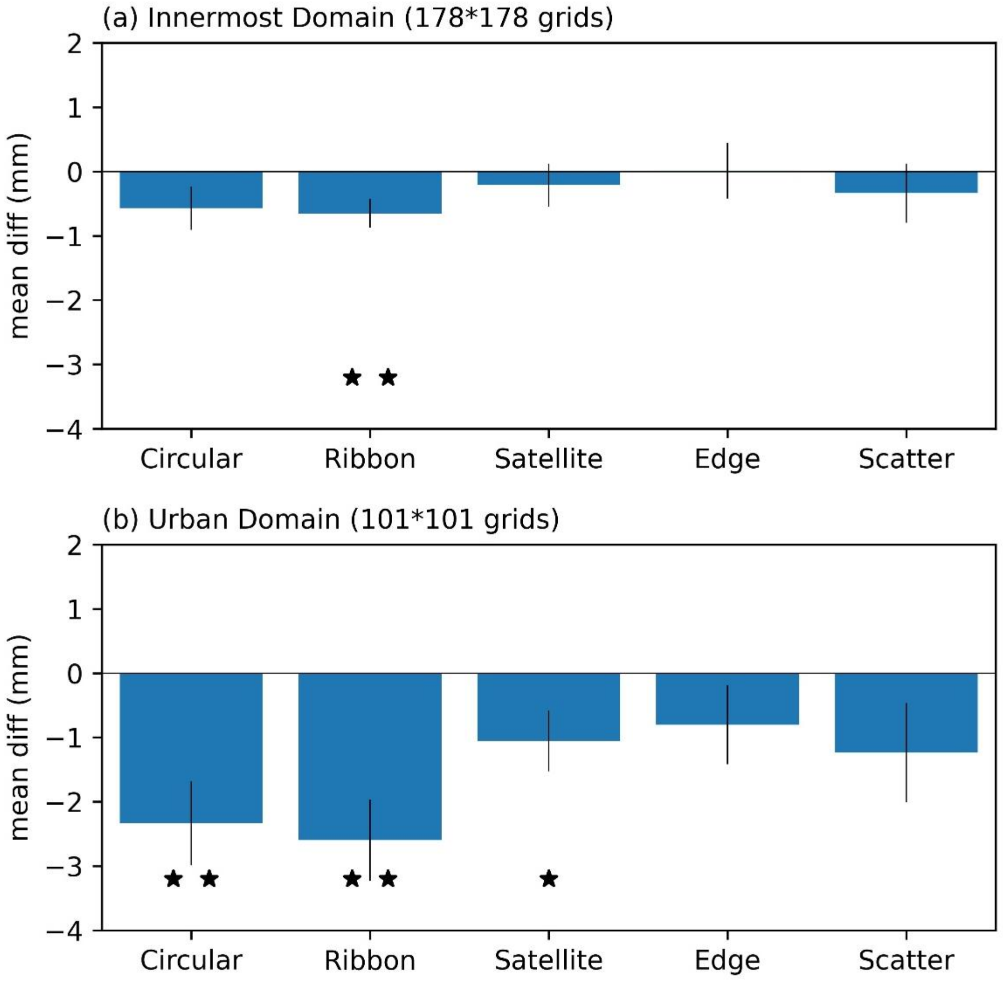

Supplementary Fig. 6 Difference in total rainfall (in mm) between each urban scenario and the "no-city" scenario. (a) the innermost domain and (b) urban domain only (i.e., 1 °×1 °). The uncertainty bar represents the standard error for the six ensemble members. "*" and "**" show statistical significance at the level of 5% and 1%, respectively, based on the Student's t-test.

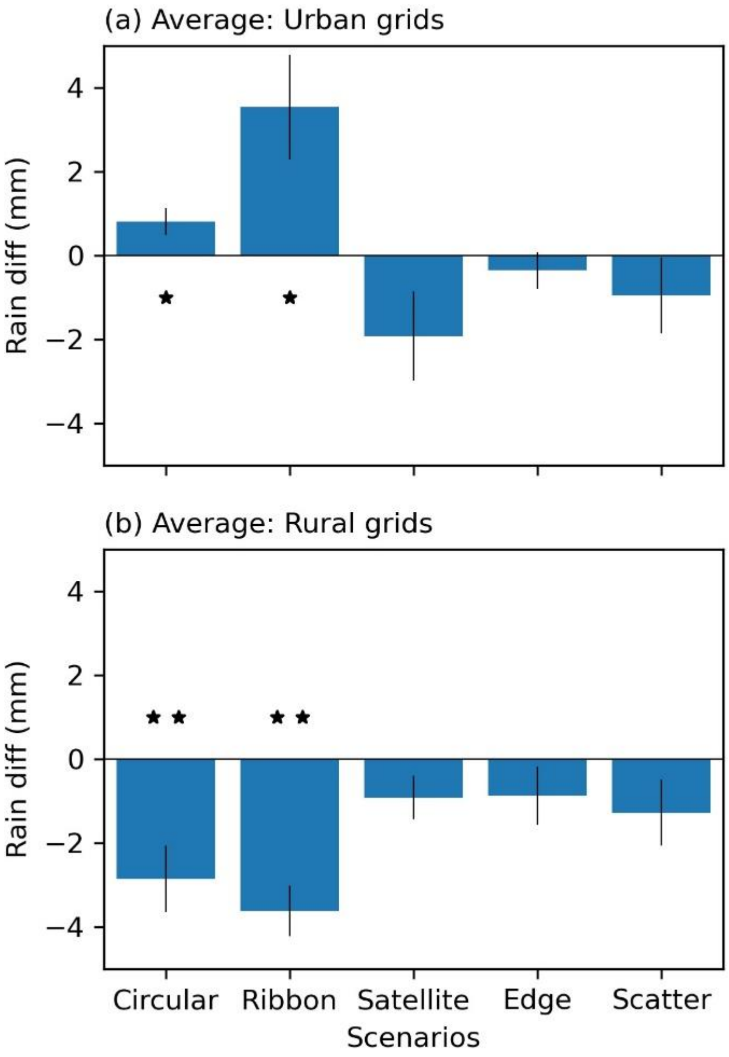

Supplementary Fig. 7 Difference in total rainfall (in mm) between each urban scenario and the "no-city" scenario. (a) urban grids and (b) rural grids. The uncertainty bar represents the standard error for the six ensemble members. "*" and "**" show statistical significance at the level of 5% and 1%, respectively, based on Student's t-test.

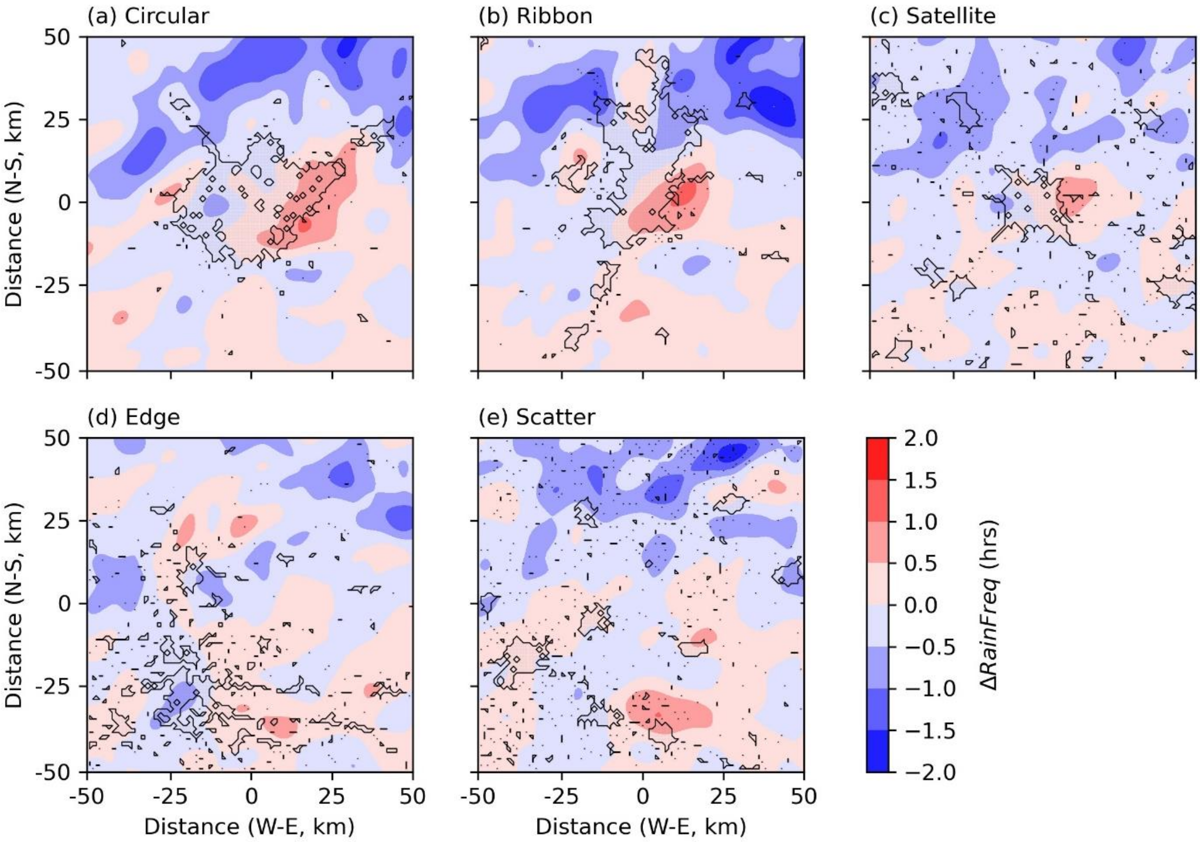

Supplementary Fig. 8 Spatial pattern of differences in extreme rainfall occurrences (i.e., hourly rain rate exceeding 2 mm/h) between each urban scenario and the "no-city" scenario. (a) Circular, (b) Ribbon, (c) Satellite, (d) Edge, and (e) Scatter. The contour highlights urban footprints.

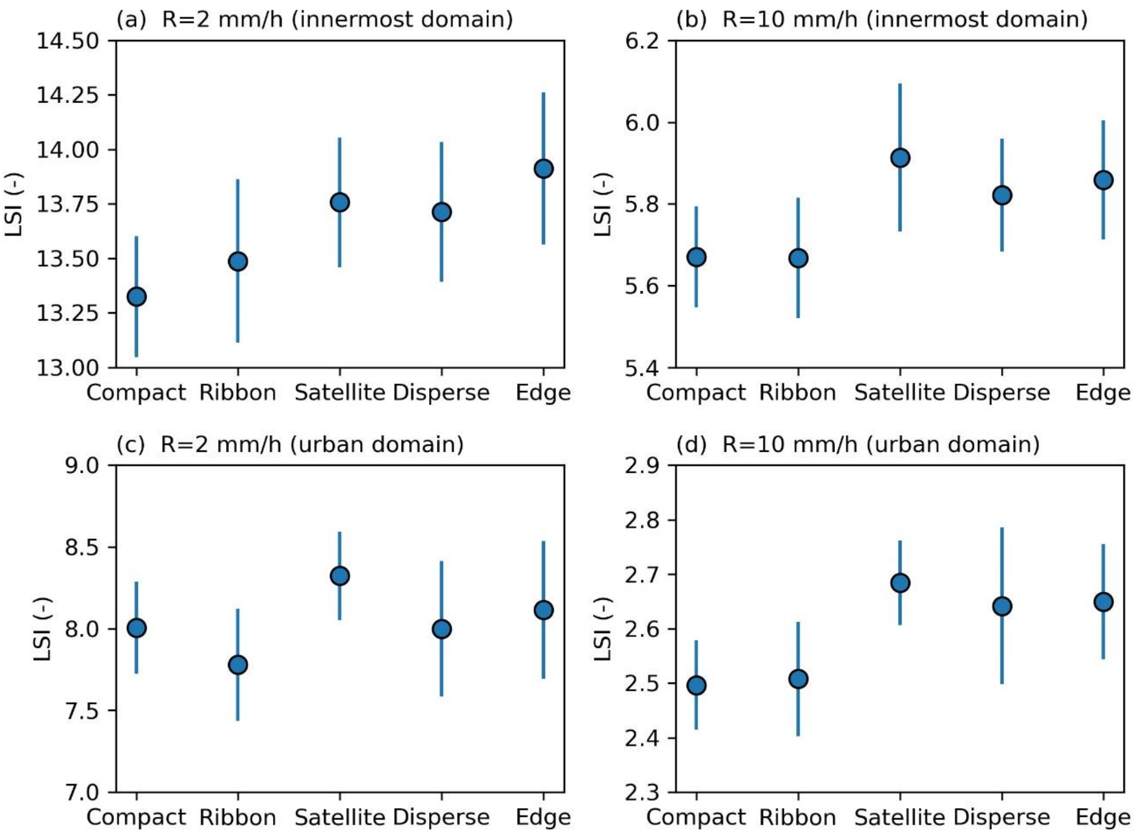

Supplementary Fig. 9 Changes in the spatial aggregation of hotspots with extreme rainfall occurrences. (a-c) extreme rainfall is defined by hourly rain rate exceeding (a, c) 2 mm/h and (b, d) 10 mm/h. The spatial aggregation is evaluated based on the landscape shape index. The evaluation is for (a, b) the innermost domain and (c, d) the urban domain only (i.e., 1 °×1 °). The scatter shows the ensemble mean, while the whisker represents the standard error of the six ensemble members.

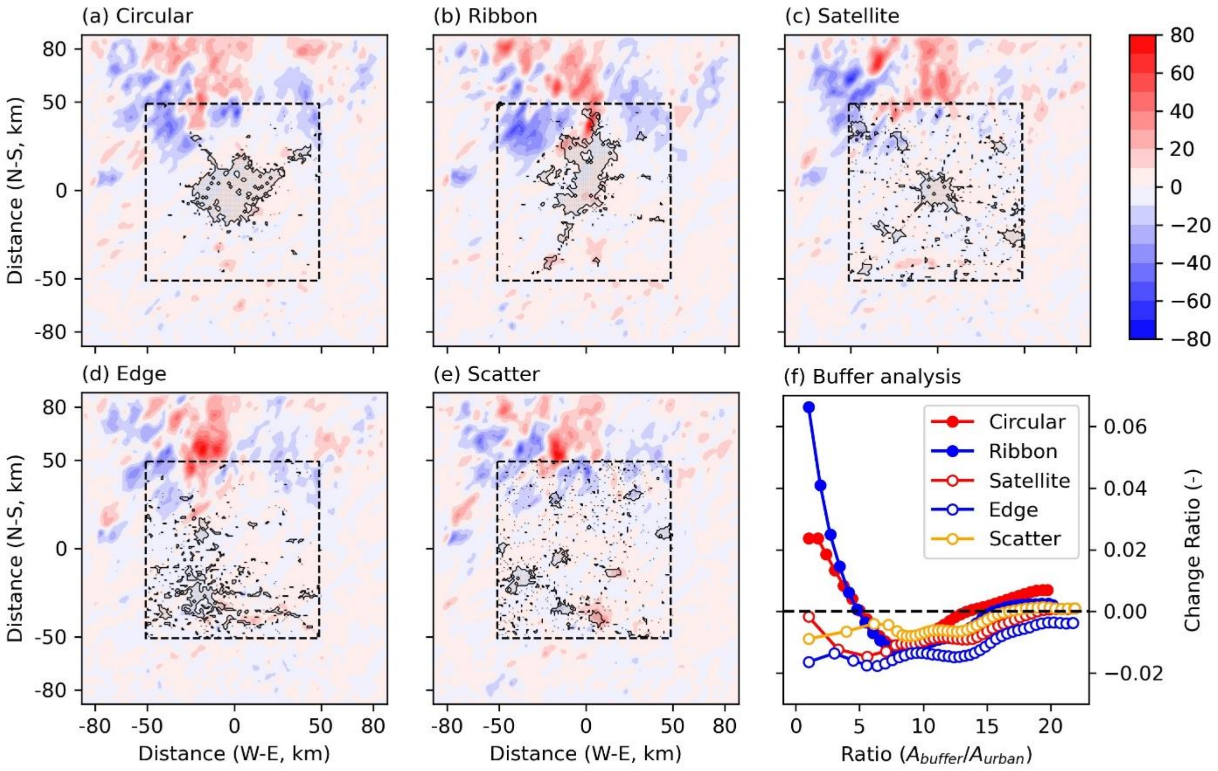

Supplementary Fig. 10 Differences in rainfall accumulation between urban scenarios and "no-city" scenario. (a-e) Spatial distribution of differences in rainfall accumulation (in mm) between each urban scenario and the "no-city" scenario; (f) the averaged change ratio within urban boundary and its buffering region. The dashed box represents the urban domain (i.e., 1 °×1 °), while the contour highlights urban footprints.

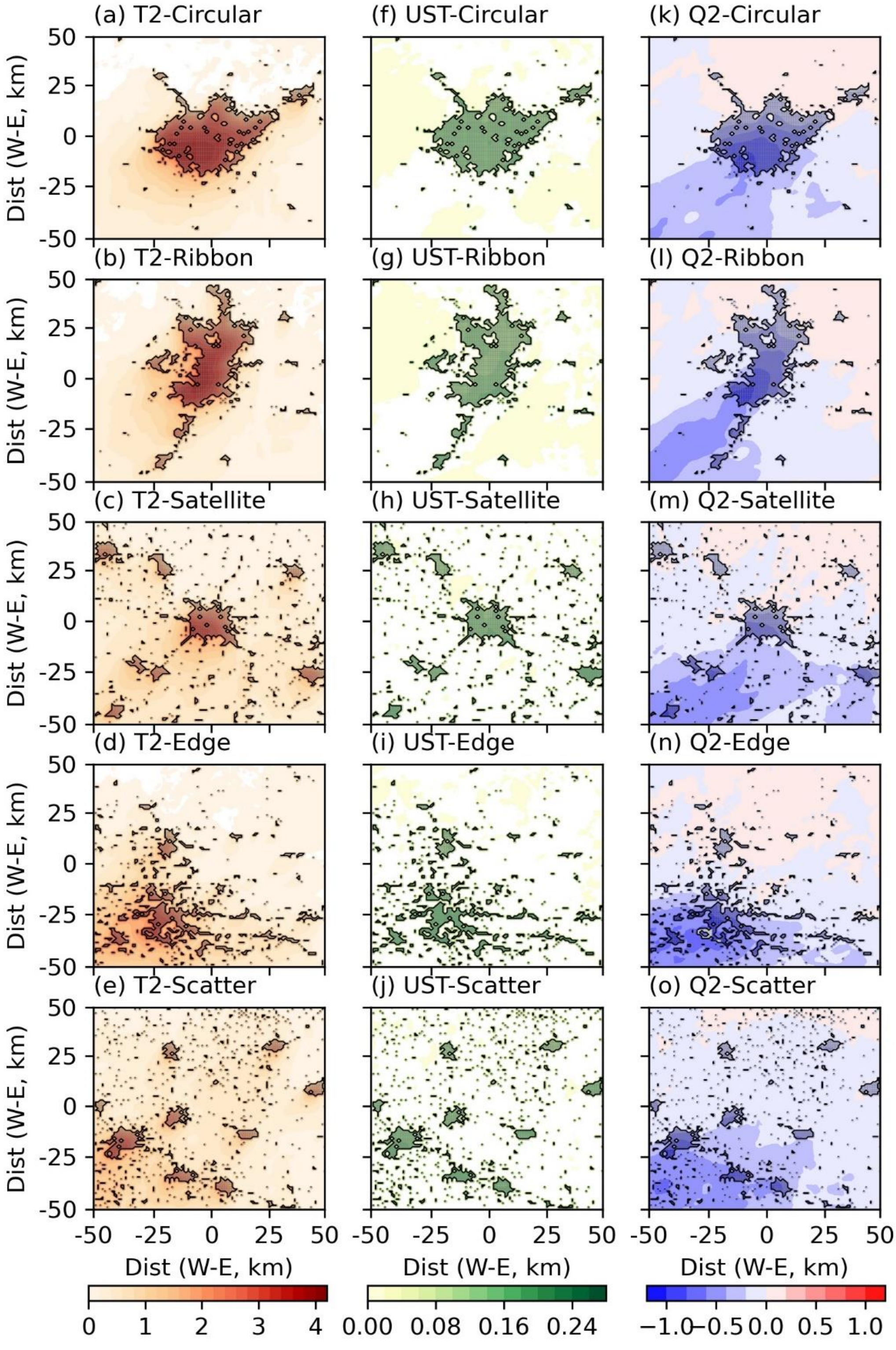

Supplementary Fig. 11 Differences in thermodynamic and dynamic variables. Difference in (a-e) 2-m temperature (in °C), (f-j) frictional velocity (in m/s), (k-o) 2-m specific humidity (in g/g) between each urban scenario and the “no-city” scenario. The black contour highlights urban footprints.

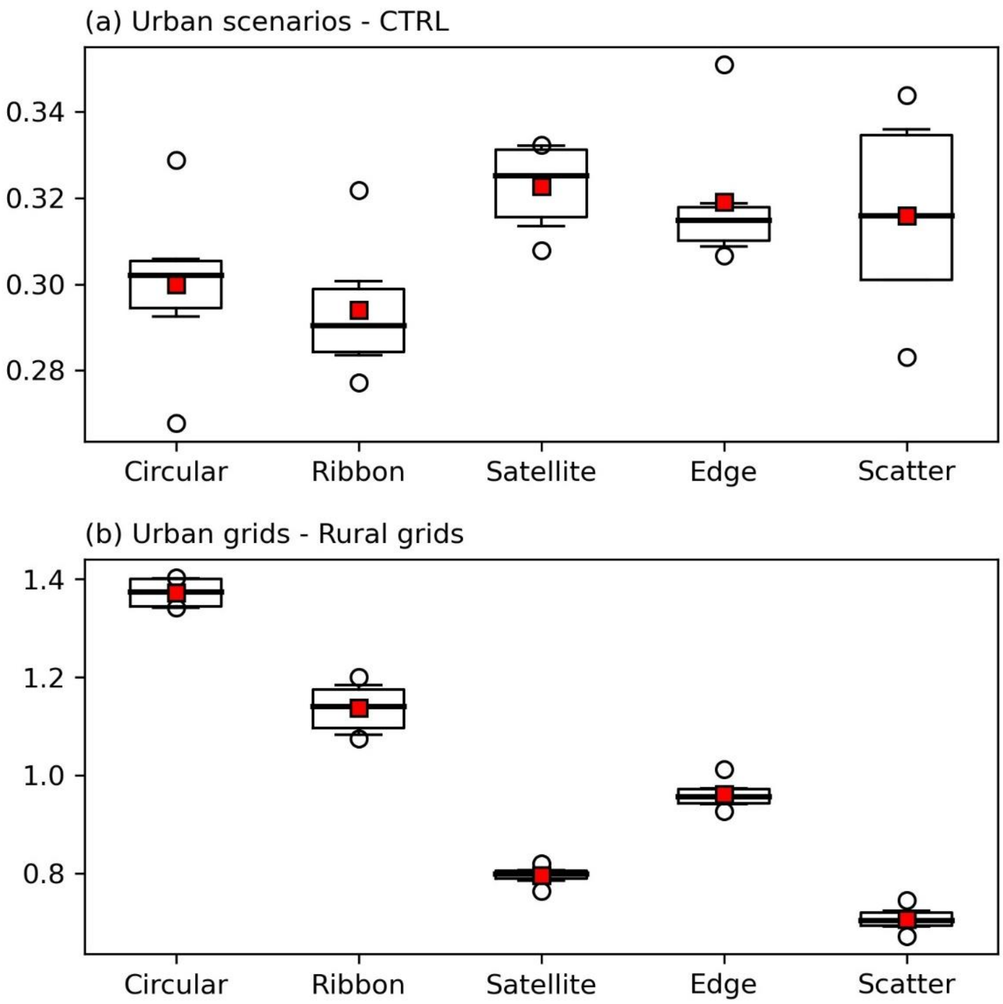

Supplementary Fig. 12 Differences in temperature across different scenarios. (a) Domain-averaged anomalies in 2-m temperature between each urban scenario and the "no-city" scenario (i.e., CTRL); (b) 2-m temperature anomalies between urban grids and rural grids for each urban scenario. The box spans the 25th and 75th percentiles, and the whiskers represents 5th and 95th percentiles. The black line represents the median values, while red squares show the mean values.

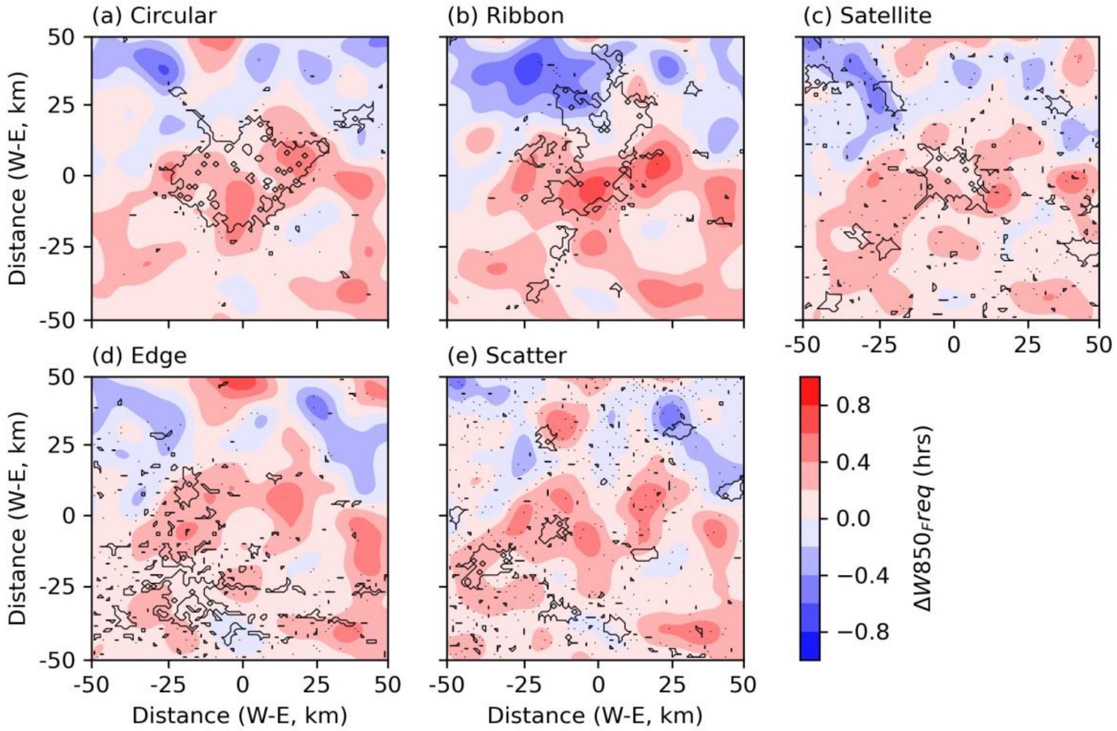

Supplementary Fig. 13 Spatial pattern of differences in the frequency of positive velocities at the level of 850 hPa. (a) Circular, (b) Ribbon, (c) Satellite, (d) Edge, and (e) Scatter. Shade represents low-level convergence, between each urban scenario and the "no-city" scenario. The contour highlights urban footprints.

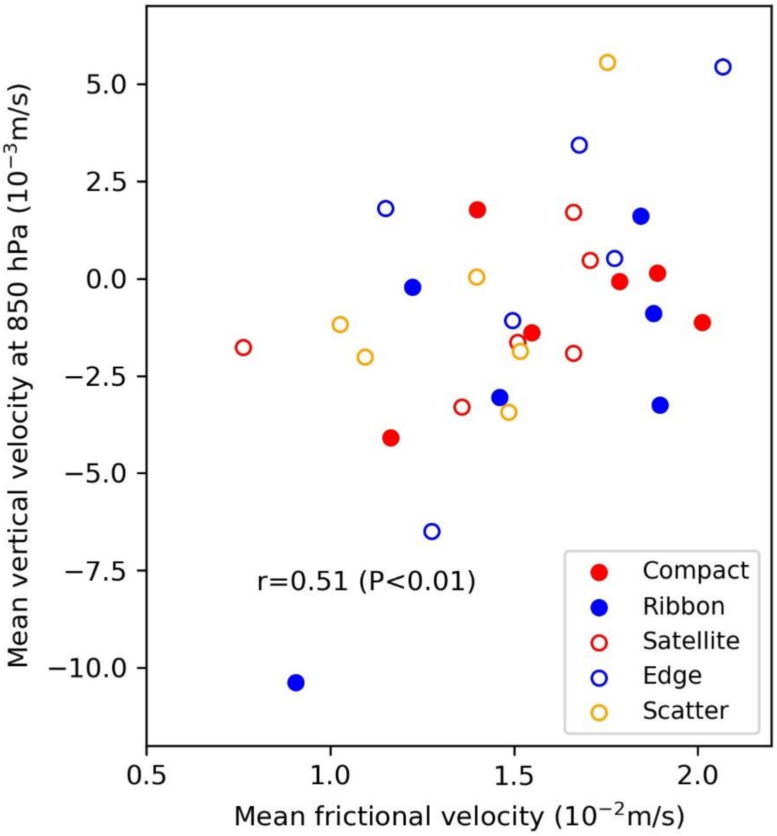

Supplementary Fig. 14 Differences in surface roughness and convergence. Relationship between the vertical velocity at the level of 850 hPa (in m/s, represented as the difference between each urban scenario and the "no-city" scenario) and mean frictional velocity (in m/s, represented as the difference between each urban scenario and the "no-city" scenario). The correlation coefficient and p-value are shown in the plot.

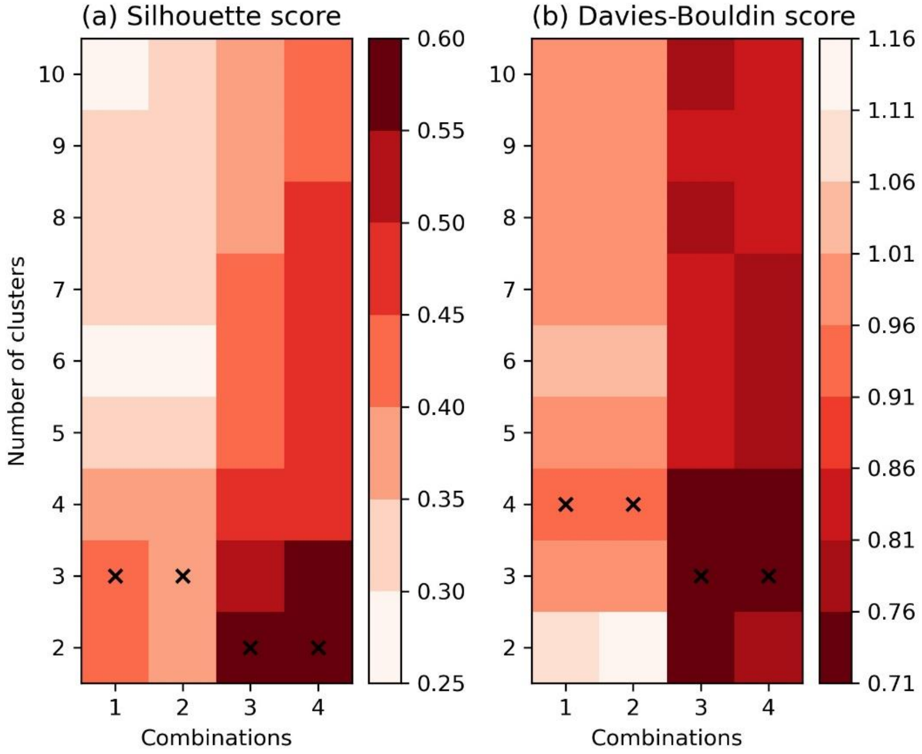

Supplementary Fig. 15 Clustering metrics. (a) Silhouette score and (b) Davies-Bouldin score for the K-means clustering algorithm using different conditions (see supplementary Table 2 for different combinations of descriptors used in clustering). The cross symbols highlight the optimal cluster number given the corresponding conditions. We choose three as the number of clusters, as it gives the most frequent optimal values.

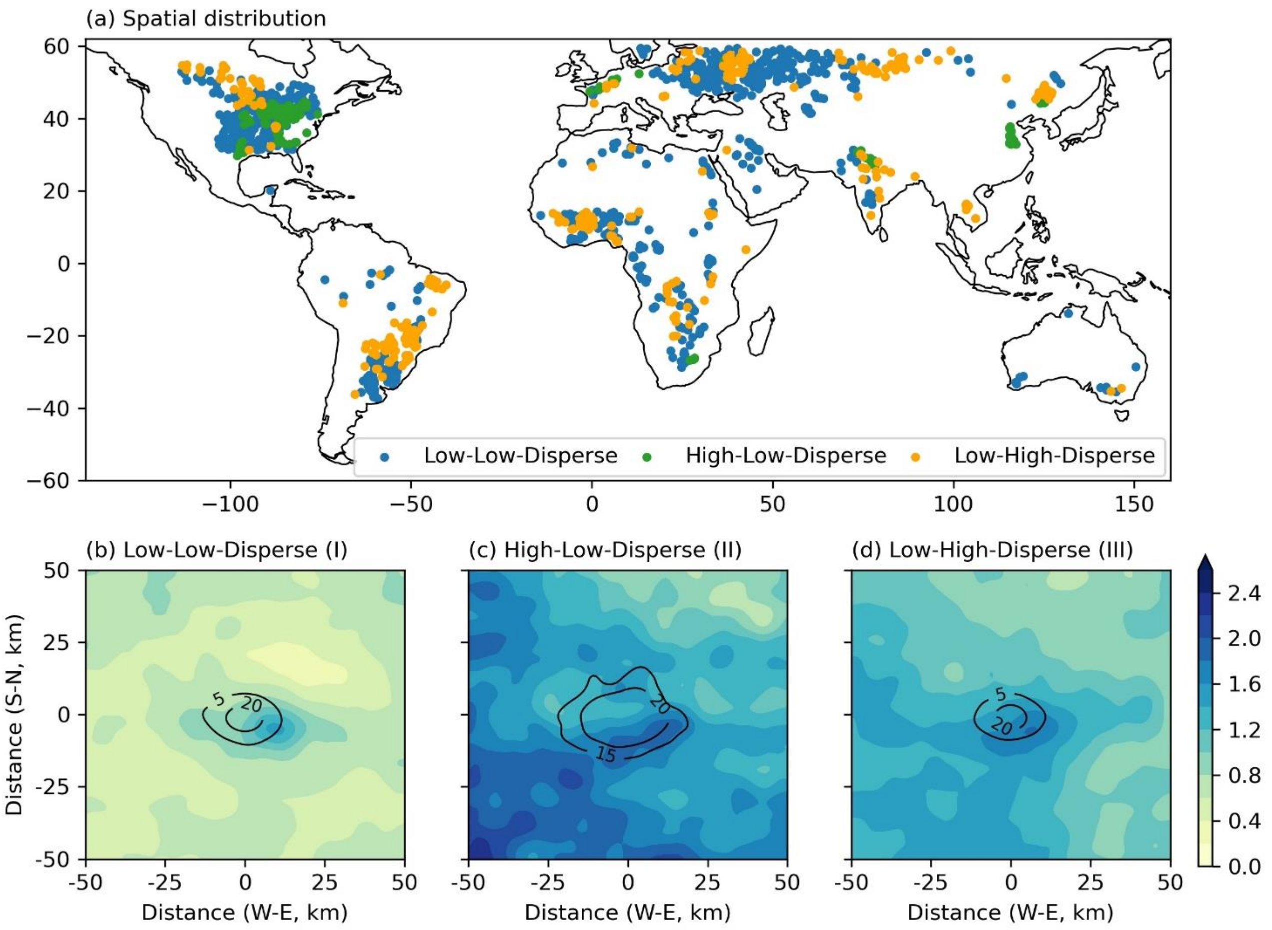

Supplementary Fig. 16 Divergent urban development patterns and the associated anomalies in spatial rainfall patterns. (a) Spatial distribution of cities with three different development patterns in a 2 °×2 ° domain centered on each city; (b-d) composite mean change ratios in extreme rainfall (i.e., exceeding the 99th percentile daily rainfall of rainy days) frequencies for different city groups with diverse development patterns between the period 2000-2005 and 2016-2020. The contour shows composite mean number of urban pixels (normalized by dividing the maximum number of urban pixels), providing an approximation of city boundary.

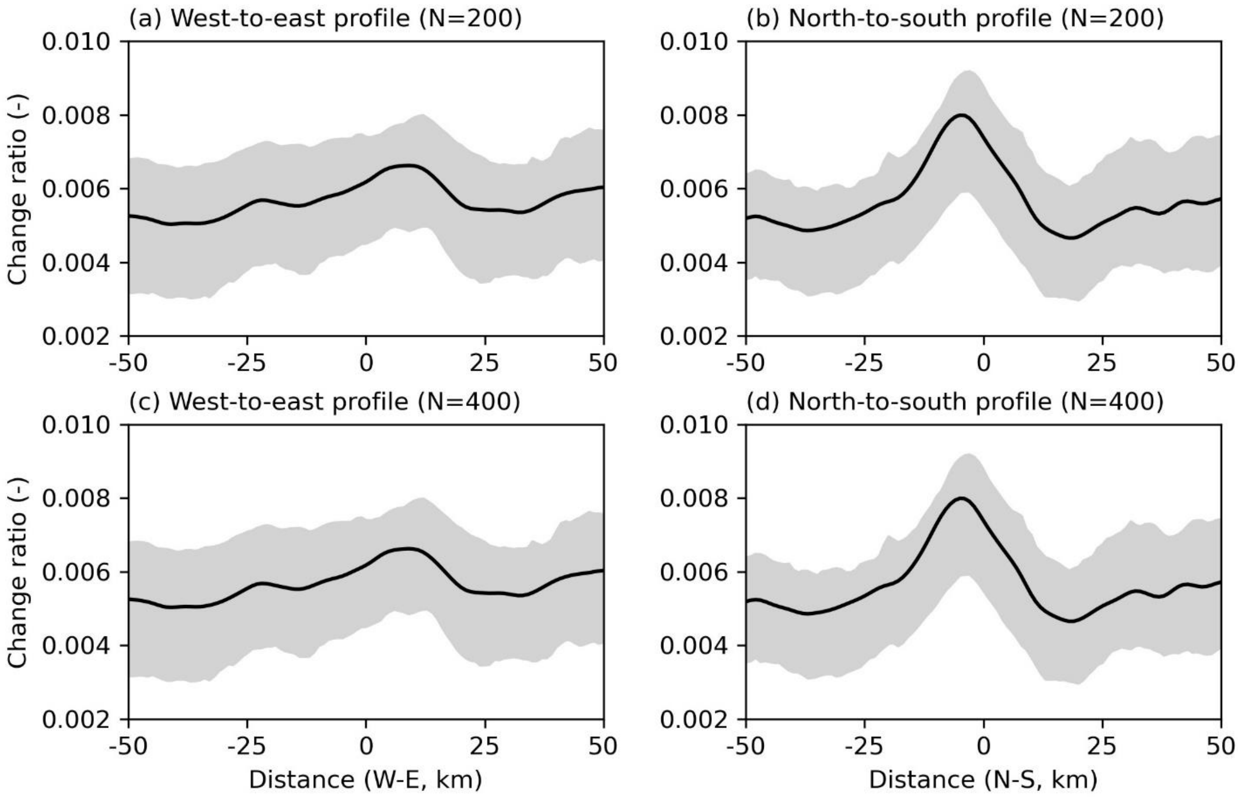

Supplementary Fig. 17 Profiles of the changes in extreme rainfall frequency for cities in Group I by randomly selecting (a, b) 200, and (c, d) 400 cities. The selection is done 100 times in total. Grey shade represents the range between 25 and 75 percentiles of the 100 composite mean profiles. Black line shows the composite mean profile for the entire group (see Figure 1b for spatial pattern). The x axis represents relatively distance (in km) from the city center.

Supplementary Tables

Supplementary Table 1. List of aggregation metrics.

**Aggregation metrics**	**Descriptions**
Landscape shape index	The sum of all edges divided by the square root of the total area, adjusted by a constant for a square standard
Number of patches	Number of patches that are isolated from each other
Patch density	Number of patches divided by total area
Splitting index	Total area of a landscape squared divided by the sum of the areas squared for each individual patch

Supplementary Table 2. Combinations of different descriptors for clustering cities into different urban development patterns. Note that: $\Delta TA=(TA_{2018}-TA_{2003})/(TA_{2018}+TA_{2003})$; $\Delta LSI=(LSI_{2018}-LSI_{2003})/(LSI_{2018}+LSI_{2003})$.

Combinations	Descriptors
1	TA_{2003}, LSI_{2003}, ΔTA, ΔLSI
2	TA_{2018}, LSI_{2018}, ΔTA, ΔLSI
3	ΔTA, ΔLSI
4	TA_{2003}, LSI_{2003}, TA_{2018}, LSI_{2018}

Supplementary Table 3. Urban canopy parameters adopted in the simulations. The urban land use category is set to industrial/commercial in the present study.

**Parameter**		**Value**
Building height (m)		10
Roof width (m)		10
Road width (m)		10
Anthropogenic heat (W m^{-2})		90
Urban fraction (-)		0.95
Heat capacity ($J\ m^{-3}\ K^{-1}$)	Roof	1.0E6
	Building wall	1.0E6
	Road	1.4E6
Thermal conductivity ($J\ m^{-3}\ K^{-1}$)	Roof	0.67
	Building wall	0.67
	Road	0.404
Surface albedo (-)	Roof	0.20
	Building wall	0.20
	Road	0.20
Surface emissivity (-)	Roof	0.90
	Building wall	0.90
	Road	0.95
Roughness length for momentum (m)	Roof	0.01
	Building wall	0.0001
	Road	0.01
